# Updating the Relationship Between the Threshold Value of Average Nucleotide Identity and Digital DNA–DNA Hybridization for Reliable Taxonomy of *Corynebacterium*

**DOI:** 10.3390/vetsci11120661

**Published:** 2024-12-17

**Authors:** Haitham Elbir

**Affiliations:** Camel Research Center, King Faisal University, 400 Al-Ahsa, Hofuf 31982, Saudi Arabia; helbir@kfu.edu.sa

**Keywords:** dromedary camel, digital DNA–DNA, *16S rRNA* gene, *Corynebacterium*, genome

## Abstract

Currently, bacterial classification at the species level relies on the 95–96% average nucleotide identity (ANI) value that is known to be equivalent to a 70% digital DNA–DNA hybridization (dDDH) value. However, during the routine identification of bacteria in the uteri of camels with a history of conception failure, we found that four out of the seven strains (2298A, 2569A, 2652, 2571B, 1103A, 2571A, and 335C) could not be assigned to any valid *Corynebacterium* species. Furthermore, a 70% dDDH value did not correspond to a 95–96% ANI value in strain 2569A. Thus, we aimed to classify these strains and explain the mechanisms underlying gene repertoire diversity and the disagreement we found between the ANI and dDDH cutoff values. For this study, we extracted information from the genomes of 150 *Corynebacterium*-type species and seven sequenced genomes of uterine *Corynebacterium* isolates. We found that the 96.67% OrthoANI value should be used in place of the generally accepted 95–96% ANI threshold in order to obtain an equivalent 70% dDDH value. Phylogenomic analysis determined the evolutionary position of each uterine strain. Then, strains 2652 and 2571B were classified as *C. camporealensis* based on the ANI value (98.44% and 98.72%) and dDDH value (85.8% and 88.5%). Strain 2569A had a 96.58% ANI and a 69.4% dDDH value and was classified as *C. urogenitale.* The strains 335C, 1103A, 2571A, and 2298A were classified as novel *Corynebacterium* based on the ANI value (77.12, 94.01%, 94.26%, and 94.03%) and dDDH value (21.3%, 54.1%, 54.9%, and 51.3%), respectively. Genes for menaquinone biosynthesis and the saturation of chains were detected in uterine strains and their closely related type strains. Gene gain predominates as a source of variation in the gene repertoire. Most of these genes are gained by horizontal gene transfer, driven by genomic islands and prophage. In summary, we refined the ANI cutoff value for an accurate diagnosis of *Corynebacterium*. Moreover, we clarified the mechanism underlying the diversity of the gene repertoire and expanded the number of *Corynebacterium* species isolated from the camel uterus.

## Simple Summary

*Corynebacterium* species can cause a wide range of infections in humans and animals. In this study, we isolated and classified *C. camporealensis*, *C. urogenitale*, and four novel *Corynebacterium* species from the uteri of camels for the first time. Through genomic analysis, we refined the average nucleotide identity cutoff value for an accurate diagnosis of *Corynebacterium*. Furthermore, we explored the mechanism underlying the diversity of the gene repertoire in uterine *Corynebacterium* strains.

## 1. Introduction

*Corynebacterium* species are ubiquitous in the environment and can be found in animal skin and mucous membranes, water troughs, and soil [1,2,3,4]. Furthermore, certain *Corynebacterium* species are associated with infections such as human diphtheria [5], caseous lymphadenitis, pyelonephritis, endometritis, and mastitis in animals [4,6,7,8,9]. Recently, it has been found that *Corynebacterium* is a common genus within the uteri of camels [10,11,12], equines [13], and cows [14,15,16]. Yet, the deep characterization of uterine *Corynebacterium* at the species level has rarely been performed. Furthermore, the impact of the infection on camel health and reproductive success is still unknown, motivating us to characterize these strains. The early classification of *Corynebacteria* relied on a certain taxonomic approach that comprised genotypic methods such as DNA–DNA hybridization (DDH), the sequencing of the *16S rRNA* gene [17], and phenotypic and chemotaxonomic characterization. In the genomic era, classification has increasingly been based on sequence similarity calculation and phylogenomic relationships, inferred from genome sequences [18]. Of the tools used to determine sequence similarity, the average nucleotide identity (ANI) was first introduced to measure the genome sequence similarity; this was then followed by the development of the digital DNA–DNA hybridization (dDDH) approach [19,20]. Currently, the 95–96% ANI species boundary cutoff range is known to be equivalent to the 70% dDDH value [21,22,23]. Notably, the ANI or dDDH values are usually calculated when a species exhibit ≥98.7% *16S rRNA* gene sequence similarity [23]. Reviewing the recent literature revealed that most *Corynebacterium* classification at the species level relies on using the 95–96% ANI value and 70% dDDH value as a cutoff. Exceptions include a study in which a *Corynebacterium* strain with a 94.11% ANI value, which is below 95–96%, was assigned to *C. amycolatum* [24]. On the other hand, a *Corynebacterium* strain with an OrthoANI value of 95.34%, which is within the cutoff range of 95–96%, was assigned to a new *Corynebacterium* species based on its dDDH value of 61.90% [25]. Recently, during our routine identification of bacteria in the uteri of camels with a history of conception failure, seven *Corynebacterium* strains were isolated. Of note, we found that strain 2569A, one of the seven *Corynebacterium* strains mentioned above, should be classified as a new *Corynebacterium* species according to the 70% dDDH cutoff value; at the same time, strain 2569A should be classified as *Corynebacterium urogenitale*, according to the current 95–96% OrthoANI cutoff value. Based on the information above, the taxonomy of *Corynebacterium* species seems to follow different molecular approaches and cutoff values for the classification of *Corynebacterium*. From an epidemiological point of view, unifying the ANI cutoff value will facilitate the tracing of *Corynebacterium* species among different niches. Furthermore, applying two different approaches, such as ANI and dDDH, will yield reliable classification results compared to using a single approach. This justifies the unification and refinement of the ANI cutoff value to correlate with the dDDH cutoff value. This will have an impact on the epidemiology of *Corynebacterium* species, help trace species, and provide more accurate classification. Therefore, the main objectives of the current study are to (1) clarify the reasons for the above inconsistent classification results through updating the relationship between ANI and dDDH, (2) characterize these camel uterine *Corynebacterium* isolates through phylogenomic and comparative genomic analyses, and (3) elucidate the impact of gene gain/loss on the gene repertoire diversity of camel uterine *Corynebacterium* species.

## 2. Materials and Methods

### 2.1. Sample Collection

A total of 7 *Corynebacterium* isolates were selected for genome sequencing from a collection of bacteria isolated previously from the uterus of a she-camel. Originally, the existing bacterial collection was not established specifically for the current study but comprised the results of the culturing of uterine samples during routine diagnosis.

The samples were collected during the routine examination of camels with a history of conception failure at the Veterinary Teaching Hospital, King Faisal University, in Al-Ahsa province, Saudi Arabia. For the sample collection, the perineum area of the camels was washed with acriflavine 0.1% disinfectant. The collection of samples from the uterus was performed using a double-guarded sterile swab (Kruuse, Langeskov, Denmark). Swabs were immediately cultured on 5% sheep blood agar plates at 37 °C for 48 h.

### 2.2. Genome Sequencing and Sequence Analysis

Extraction of the total genomic DNA from *Corynebacterium* isolates was performed using the Wizard genomic DNA purification kit (Promega Biotech AB, Madison, WI, USA) according to the manufacturer’s instructions. DNA libraries for genome sequencing were prepared using the TruSeq Nano DNA Library Preparation Kits (Illumina, San Diego, CA, USA). All constructed libraries were pooled and sequenced by Macrogen Inc. (Seoul, South Korea) on Illumina’s NovaSeq platform to create 2 × 151 bp paired-end reads. Following sequencing, the quality of sequence reads was assessed by Fastqc 0.11.8 [26], and low-quality bases were trimmed using fastp software version 0.20.0 [27]. Sequence reads were de novo assembled using SPAdes 3.15.4 [28]. Only scaffolds with a coverage value above 10% and a length exceeding 500 base pairs were maintained for downstream analysis.

Information regarding the open reading frames (ORFs), tRNA, and ribosomal RNAs was obtained using Prokka 1.14.6. The functional characterization of ORFs was obtained using the online BlastKOALA web server of the KEGG (Kyoto Encyclopedia of Genes and Genomes) database [29]. If no hit exists, a search against the non-redundant (NR) database was performed using BLASTP with an *E*-value of 10^3^ coverage of 70% and a similarity percent of 30% on both the query and hit.

The detection of prophages in the genomes was performed using PHASTER (Phage Search Tool Enhanced Release) [30]. It uses a scoring technique to classify prophage regions as intact (>90), questionable (70–90), or incomplete (<70). The prediction of clustered regularly interspaced short palindromic repeats (CRISPRs) was performed using CRISPRCasFinder v 1.1.2 software [31,32], which are used to accurately detect CRISPR-Cas subtype, number of CRISPRs, and spacers. As for the detection of potential virulence genes, we conducted homology searching via the BLAST tool against the virulence factor database (VFDB) [33]. Iron-related protein families were detected (heme iron transport, iron transport, siderophore synthesis, siderophore transport, transcriptional regulation, and iron storage) using FeGenie v.1.0 [34].

In general, genomic islands (GIs) consist of foreign DNA fragments gained by horizontal transfer. GIs were detected using the IslandViewer 4 web server, which is currently the most reliable approach for GI prediction [35]. The IslandViewer 4 tool integrates four different genomic island prediction methods: IslandPick, IslandPath-DIMOB, SIGI-HMM, and Islander. The default settings of IslandViewer 4 were used. The genes found in the detected GI regions were generally considered horizontally transferred genes.

### 2.3. Synteny Analysis

The genomes of uterine *Corynebacterium* and their closest type species genome were used as inputs for Mauve Contig Mover functionality to illustrate synteny blocks between two pairs of genomes [36]. The order of orthologous proteins from the BLASTP was manually investigated in Microsoft Excel. The regions covered by genes from both pairs of genomes were defined as synteny blocks, whereas regions not covered by genes were defined as synteny breaks. IslandCompare v1.0 [37] was used for the visualization of synteny breaks and the comparison of genomic islands between strains.

### 2.4. Correlation Among dDDH and OrthoANI

For the identification of clinical isolates and reclassification of type species, dDDH and OrthoANI were used for genomic similarity measurement among closely related species. All pairwise ANI values of the tested species were estimated using the command-line version of OrthoANI (OAT v. 1.40 using blastn 2.13.0+) [19] with default parameters. All pairwise dDDH values were estimated using the online (http://ggdc.dsmz.de/distcalc2.php, accessed on 22 August 2022) Genome-to-Genome Distance Calculator server (GGDC 3.0) and formula 2 for distance calculations [20]. The GC content differences of every closely related pair of species were also estimated from their genomes during the dDDH calculation by GGDC 2.1 Calculator. Two strains were considered as the same member of a species if their dDDH value was higher than 70% or if their OrthoANI value was within 95–96% [23]. When genome pairwise comparison gave less than 80% ANI values, such genomes were considered divergent and were excluded from correlation analysis among dDDH and ANI. The values for correlation analysis between OrthoANI and dDDH were calculated by statistical packages in Microsoft Excel v 16.81. Coefficients of determination (R2) among dDDH and OrthoANI were investigated by logarithmic, linear, and exponential regression analysis.

### 2.5. 16S rRNA Gene Analysis and 16S rRNA Phylogenetic and Phylogenomic

From the list of prokaryotic names with standing in nomenclature, we collected type strain names for all *Corynebacterium* species with a validly published name as of 19 July 2023. Then, we retrieved their *16S rRNA* gene sequences from their genome sequences using the PROKKA v1.14.6 annotation tool. Some species with a validly published name, such as *C. evansiae*, were excluded from the analysis as having *16S rRNA* gene sequence length shorter than 1350 nt. In addition, the *16S rRNA* gene sequence was not found for *C. beticola*. After extraction, all *Corynebacterium* species and *16S rRNA* gene sequences with *Mycobacterium tuberculosis* H37Rv were individually aligned using the MAFFT v7.490 program with default parameters [38]. Maximum likelihood phylogeny was computed in IQ-TREE v2.2.0 [39] using the best-fit model GTR+F+I+G4, defined by the software according to the Bayesian information criterion scores. The ultrafast bootstrap analysis was based on 1000 replicates and the constructed phylogeny was visualized using online iTOL v 7 software. For the phylogenomic tree, a sum of 152 genomes from type *Corynebacterium* species with validly published names was retrieved from the GenBank database. To maintain the same annotation, all the downloaded genomes were annotated using PROKKA v1.14.6. For phylogenomic tree inference, only nucleotide sequences of single-copy orthologous core genes (SOCGs) shared by all analyzed genomes of *Corynebacterium* species were used. Determination of those common SOCGs was performed by utilizing GET_HOMOLOGUES scripts [40] using a sequence similarity of >30%, a query coverage of >70%, and an E-value of >1 × 10^−5^. The analysis was based on protein sequence, which yielded 244 SOCGs. The nucleotide sequence was used instead of the protein sequence to better distinguish between closely related species. Subsequently, each SOCG was aligned using MAFFT v7.490, and resulting alignments were concatenated and used for maximum likelihood tree inference with IQ-TREE v2.2. Branch support was assessed by 1000 replicates for ultrafast bootstrap analysis. The best-fitting model of sequence evolution determined by ModelFinder that was implemented in IQ-TREE v 2.2.0 was used. The tree was visualized using online iTOL v 7 software. Finally, pairwise *16S rRNA* gene sequence similarities between species were determined by the BLASTN method.

### 2.6. Inference of Gene Gain and Loss

BadiRate v1.35 [41] was used to estimate gain and loss events, as well as turnover rates of gene families only for genomes located in the same phylogenetic clades encompassing uterine *Corynebacterium* isolates. The BadiRate analyses were run using the Gain and Death (GD) model [36]. The GD rates of gene families were determined using a branch-free rates model (GD-FR-ML) supposing that each branch has its own turnover rates. BadiRate analyses require an ultrametric tree of the taxa examined; hence, we used the ete v 3.1.3 Python package [42] to convert our supermatix ML tree into an ultrametric tree. The results from GET_HOMOLOGUES scripts and the ultrametric tree obtained from our estimated phylogenomic ML tree were used as inputs for BadiRate.

## 3. Results

### 3.1. Identification Based on 16S rRNA Gene Sequence

**Figure 1:**
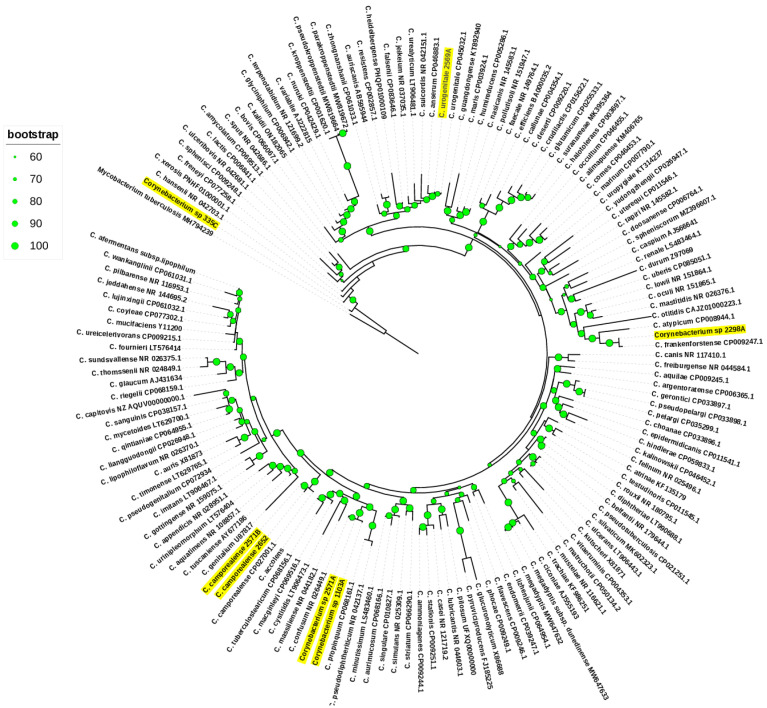
*16S rRNA* phylogenetic tree. The green circles above the branches are bootstrap support values. Uterine *Corynebacterium* isolates are highlighted in yellow color.

The strains 2652, 2571B, 2569A, 1103A, 2571A, 2298A, and 335C were initially assigned to the genus *Corynebacterium* based on the *16S rRNA* gene sequence similarities. Strains 2652 and 2571B were grouped with the type species *C. camporealensis* DSM-44610 (99.15 and 99.67% *16S rRNA* gene sequence identity) as mentioned in the *16S rRNA* phylogeny tree (Figure 1). Strains 1103A and 2571A were grouped with *C. confusum* DSM 44384 (99.74 and 99.74% sequence identity). The strain 2569A was grouped with *C. urogenitale* DSM 108747 (99.54% sequence identity). The strain 2298A was grouped with *C. frankenforstense* DSM 45800 (99.28% sequence identity). The remaining isolate 335C emerged as a separate branch, sharing 98.16% *16S rRNA* gene sequence similarity to the *C. hansenii* DSM 45109 type strains, which was below the 98.65% similarity threshold value used to define new species. Therefore, one putative novel species of *Corynebacterium* might be suggested based on the *16S rRNA* gene sequence. To investigate the usefulness of *16S rRNA* gene sequence similarity for the identification of *Corynebacterium* isolates to the species level, we examined the pairwise similarity values of this gene for *Corynebacterium* validly published species. A pairwise sequence comparison revealed that 22 pairs of species exceeded the 98.7% cutoff values for species delineation (Supplementary Table S1).

### 3.2. Identification Based on Genome Sequence

**Figure 2:**
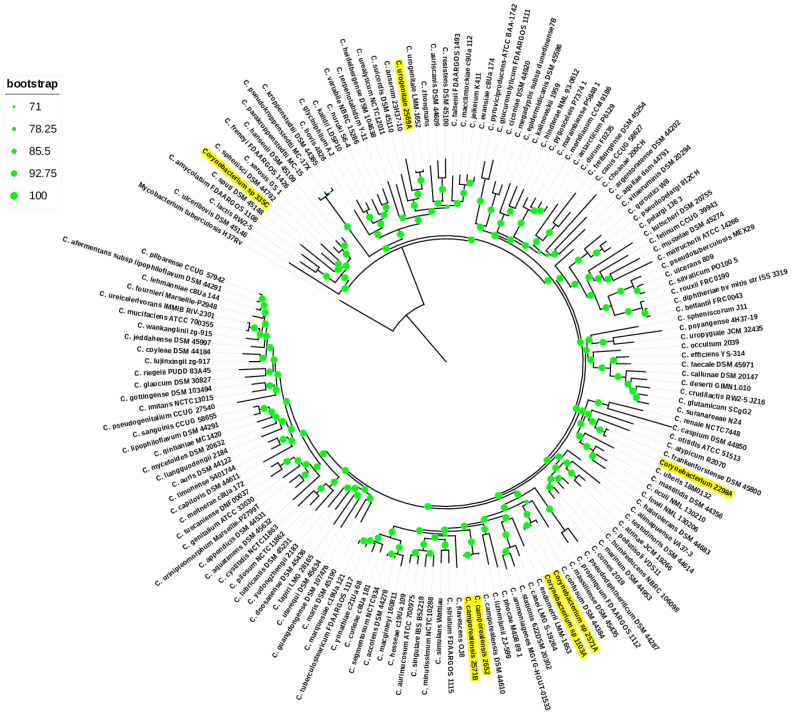
Phylogenomic tree inferred from core genes. The green circles above the branches are bootstrap support values. Uterine *Corynebacterium* isolates are highlighted in yellow color.

The first whole genome-level approach to cluster the uterine isolates was the core-genome phylogenetic analysis. A phylogenomic tree based on 244 single-copy orthologous core genes clearly clustered each uterine isolate to its closest type species and was supported by a high bootstrap value (Figure 2). Noteworthy, the topology of the phylogenomic is similar to the above *16S rRNA* phylogenetic tree of the uterine isolates, except for the strain 335C, which clustered with *C. sphenisci* DSM 44792 in the phylogenomic tree. Secondly, we computed ANI and dDDH values for each clinical isolate with that of its closest type strain. The ANI values between the type strain *C. camporealensis* DSM-44610 and strains 2652 and 2571B were 98.4% and 98.7%, respectively; thus, they were above the similarity threshold value (94–96%) used to define new species. In contrast, their dDDH values were 85.8% and 88.5% above the 70% similarity threshold value, thereby confirming the assignment of strains 2652 and 2571B to *C. camporealensis*. The ANI values between the type strain *C. confusum* DSM 44384 and the strains 1103A and 2571A were 94.01% and 94.26%, close to the similarity threshold value (95–96%), and their dDDH values were 54.1% and 54.9%, far from the 70% dDDH value; thus, they were not assigned as *C. confusum* species. The ANI values between the strains 2298A and the closest related *C. frankenforstense* DSM 45800 were 94.03%, close to the similarity threshold value (95–96%), and their dDDH values were 51.3%, far from the 70% dDDH value; thus, they were not assigned to *C. frankenforstense* species. The ANI values between the strains 2569AC and the closest related *C. urogenitale* DSM 108747 were 96.58% above the similarity threshold value (95–96%), and their dDDH values were 69.4%, close to the 70% dDDH value, thereby confirming the assignment to *C. urogenitale* species. The 77.12% ANI and 21.3% dDDH values between the strain 335C and the most closely related species *C. sphenisci* DSM 44792 were below the recommended cutoff values of 95–96% ANI and 70% dDDH for describing new species within the genus, thereby indicating that this isolate at the genome level constitutes a novel *Corynebacterium* species.

Finally, the menaquinones are a type of bacterial respiratory quinones that are considered a taxonomic marker according to the length and level of saturation in their isoprenoid chains. The existence of genes involved in menaquinone biosynthesis and elongation of isoprenoid side chains was examined. All uterine *Corynebacterium* strains and their related type strains contain the menaquinone biosynthetic genes (*menA*, *menB*, *menC*, *menD*, *menE*, *menF*, and *menG*), a menaquinone chain elongation gene (*uppS* and *hepST*), and a menaquinone chain saturation *menJ* gene. Therefore, the menaquinone of uterine strains is expected to be saturated due to the presence of the *menJ* gene.

### 3.3. Correlation Between dDDH Values and ANI

**Figure 3:**
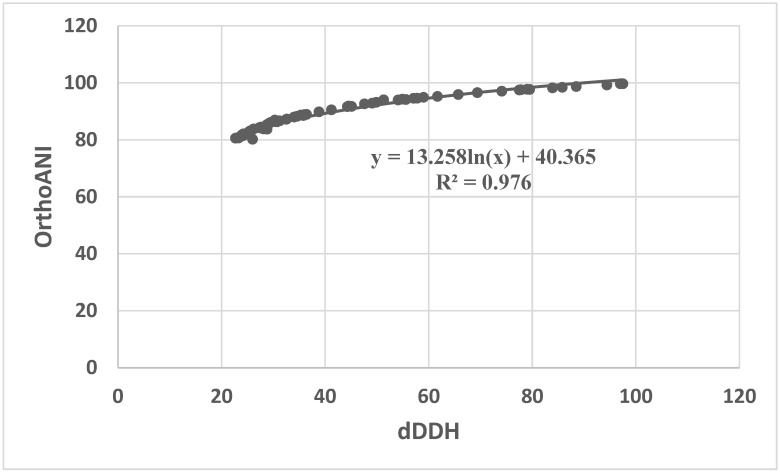
Correlation analysis between OrthoANI and dDDH from the pairs of *Corynebacterium* species.

We first computed the dDDH and OrthoANI values between all *Corynebacterium* type species whose genomes are sequenced. Then, the OrthoANI values between all pairs of strains above 80% were only included for correlation analysis with dDDH values. The results indicated that in addition to strain 2569A and *C. urogenitale* DSM 108747, there were two species pairs, i.e., *C. diphtheriae* bv mitis str ISS3319: *C. belfantii* FRC0043 and *C. ureicelerivorans* IMMIB RIV-2301: *C. mucifaciens* ATCC 700355, in which the dDDH values were below 70%, but the OrthoANI values were within 95~96% (Table S1). So, the above data also provide conflicting classification results. Notably, ANI and dDDH values between the type strain *C. segmentosum* NCTC934 and *C. accolens* DSM 44278 were 97.1% and 74.1%; therefore, they were above the 95–96% ANI and 70% dDDH similarity threshold value used to define new species. In addition, they cluster together on the core genome and *16S rRNA* gene tree. Thus, at the genomic and phylogeny level, they belong to the same species, which indicates misidentification. Finally, we estimated the correlation between dDDH values and ANI via different regression models. Regression analysis between dDDH and ANI values revealed that ANI strongly correlates with dDDH using the logarithmic model with r2 correlation values (0.98) followed by linear and exponential models with r2 correlation values (0.9 and 0.88). According to our results, the standard cutoff point of 70% dDDH similarity for species demarcation corresponded to an ANI of 96.67% in the *Corynebacterium* genus (Figure 3).

### 3.4. Genome Content and Gene Dynamics

The general genome features of uterine *Corynebacterium* isolates are shown in Table 1. In brief, strains 1103A, 2571A, and 2298A differ from type strain by 12.3%, 18.3%, and 10.2% of their total genes, respectively. *C. urogenitale* 2569A differed from its type strain by 11.3% of its total genes. *C. camporealensis* 2652 and *C. camporealensis* 2571B differed from their type strain by 8.3% and 8.3% of their total genes.

**Figure 4:**
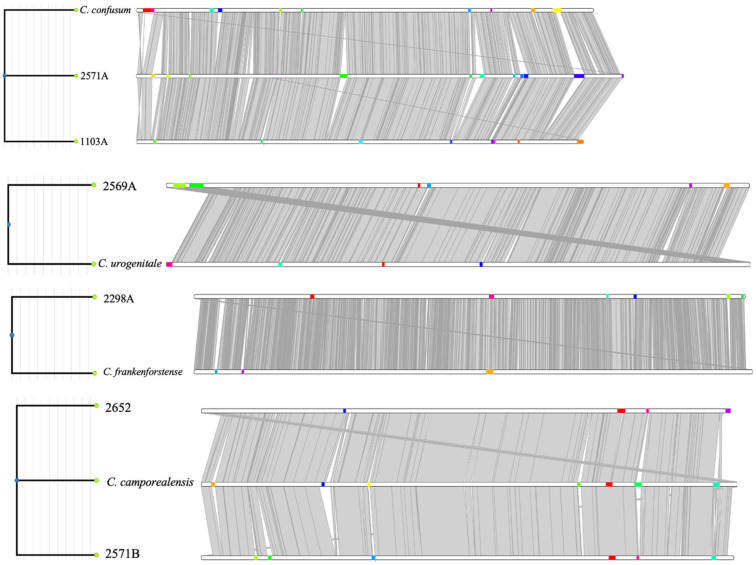
GIs are shown on the linear shape of the genome as colored blocks and GIs coloured by cluster. Genomes are represented by linear white bars, whereas gray indicates genome alignments.

We found a diverse pool of mobile genetic elements, including genomic islands (MGEs) (Table 2) and intact or remanent prophages (Figure 4). The uterine strains also harbored various types of defense systems against MGEs, including type III and type I RM systems and CRISPR-Cas system subtype I-E, with spacers that reflect their previous exposures to phages. Notably, we found a that percentage of 38.7%, 64.70%, and 49.3% of strain-specific genes were associated with the pool of MGEs in *C. confusum* DSM 44384 and strains 1103A and 2571A, respectively. In the case of *C. camporealensis* DSM-44610, *C. camporealensis* 2652, and *C. camporealensis* 2571B, we found that percentages of 55.1%, 20.6%, and 51.9% of strain-specific genes were associated with the pool of MGEs. As for *C. urogenitale* DSM 108747 and strain 2569A, we found that percentages of 46.2% and 22.32% of strain-specific genes were associated with the pool of MGEs. As for strain *C. frankenforstense* DSM 45800 and 2298A, we found that percentages of 13.6% and 19.9% of strain-specific genes were associated with the pool of MGEs.

The pairwise comparison of species genomes can yield synteny maps that allow for the examination of conserved genomic regions and evolutionary events across different species. Here, we have used the closed genome of the type species to order the contigs of uterine *Corynebacterium* isolates. Following this procedure, we found large locally collinear blocks between the type species and the related uterine *Corynebacterium* isolates, which indicate high synteny. This synteny was disrupted by the presence of several regions of differences that represent the above-mentioned strain-specific genes.

**Figure 5:**
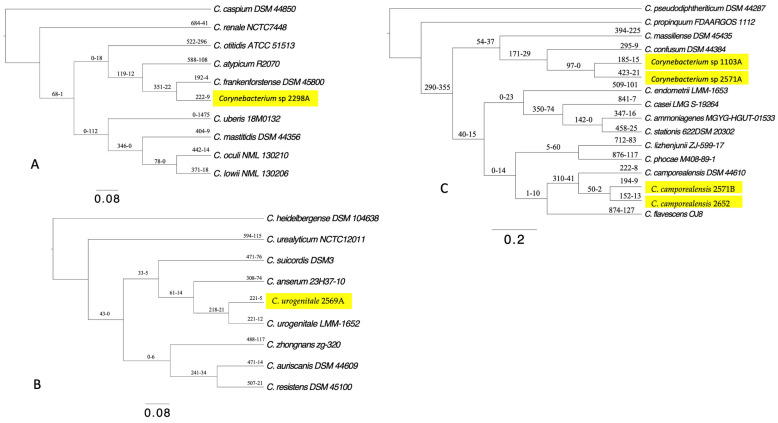
BadiRate analyses results according to branch-free rates model (GD-FR-ML). The numbers on the branches indicate the minimum number of gains (number before the dash) and the minimum number of losses (number after the dash). (**A**) Subfigure A shows gene turnover for *Corynebacterium* sp. 2298A highlighted in yellow color. (**B**) Subfigure B shows gene turnover for *C. urogenitale* 2569A. (**C**) Subfigure C shows gene turnover for *C. camporealensis* 2652 *C. camporealensis* 2571B, *Corynebacterium* sp. 1103A, and *Corynebacterium* sp. 2571A.

Finally, to examine the dynamics of gene content differences in more detail, we investigated gene gain/loss (turnover) events for each uterine *Corynebacterium* strain through the phylogeny via BadiRate v1.35 software. The results indicated that the number of genes gained dominates over gene loss in all species, although the number of gene gains or losses is comparable with species. The strain 2571A showed a (gain = 423, loss = 21), which is the greatest gene turnover (gene gain and loss) among other species. In contrast, the two strains of *C. urogenitale* from the uterus of cattle and camel showed a similar turnover for gene gain with a slight difference in gene loss (Figure 5).

### 3.5. Comparison of Functional Traits 

To go in depth into the functional profile of uterine *Corynebacterium* strains, the metabolic KEGG modules and transporters were reconstructed for each genome. The emphasis was on the selection of complete genetic traits instead of the existence of individual genes. A detailed analysis of the traits for *C. camporealensis* DSM 44610 (sheep milk isolate), *C. camporealensis* 2571B (camel uterine isolate), and *C. camporealensis* 2652 (camel uterine isolate) revealed that they harbored similar types of PTS and ABC transporters, except strain 2571B, which harbored the two genes (*irtA* and *irtB*), encoding an ABC transporter for iron import. Also, a total of 55 metabolic KEGG modules were shared between *C. camporealensis* 2571B and *C. camporealensis* 2652 and the related type species *C. camporealensis* DSM 44610. As for the differences in metabolic potentials, the module M00098 for Acylglycerol degradation was specific for *C. camporealensis* 2652.

In the case of camel uterine *C. urogenitale* 2569A and *C. urogenitale* DSM 108747 (cow uterus isolate), the detailed analysis of the traits revealed that the *C. urogenitale* 2569A strain differed from the related type strain *C. urogenitale* DSM 108747 by harboring a taurine transport system encoded by the *tauACB* genes. Notably, *C. urogenitale* strains lack enzyme I and phosphocarrier protein HPr. Therefore, they lack the PTS system. Moreover, a total of 56 modules were shared between *C. urogenitale* 2569A and the related type strain *C. urogenitale* DSM 108747. As for the differences in metabolic potentials, the module M00083 for fatty acid biosynthesis and elongation was specific for *C. urogenitale* 2569A. As for camel uterine strain 2298A and its related type strain *C. frankenforstense* DSM 45800 (cow milk isolate), both harbored similar types of PTS and ABC transporters, except that the *C. frankenforstense* DSM 45800 genome harbored the genes *RbsA*, *RbsB*, and *RbsC* for the ribose ABC transporter. In contrast, the genome of strain 2298A harbors the *musEFG*/*msmX* genes for the maltose ABC transporter. In addition, a total of 53 modules were shared between strain 2298A and its related type species *C. frankenforstense* DSM 45800. As for the differences in metabolic potentials between the two strains, the modules M00125 and M00970 for riboflavin biosynthesis and proline degradation were specific for strain 2298A. As for the differences in ABC transporters between the strains 2571A and 1103A (camel uterine isolate) and their related type strain *C. confusum* DSM 44384 (human plantar abscess), the *C. confusum* DSM 44384 harbored the iron import ABC transporter encoded by *irtAB* genes, heme ABC transporter encoded by *hmuUV* genes, and the glycine betaine ABC transporter encoded by *proVWX* genes. 

Another strain specific ABC transporter is the glutathione uptake system carried by strain 2571A. Other specific ABC transporters carried by strain 1103A were iron–siderophore transporters encoded by *fepBDGC* genes and the potassium uptake system encoded by *KtrAB* genes. As for the unshared PTS transporter, the β-glucoside PTS transporter is missing from strain 1103A but is present in *C. confusum* DSM 44384 and 2571A. Notably, beta-glucosides, such as cellobiose, result from the hydrolysis of plant cellulose; therefore, decaying vegetation can act as a source of carbon for soil bacteria. This suggests that strain *C. confusum* DSM 44384 and strain 2571A have the potential to survive in soil via the utilization of decaying vegetation as a carbon source. In addition, a total of 55 metabolic KEGG modules were shared between strains 2571A and 1103A and *C. confusum* DSM 44384. As for the differences in metabolic potentials between strains 2571A and 1103A and *C. confusum* DSM 44384, the two modules, M00551 and M00568, for aromatic compound degradation were specific for strains 2571A and 1103A. One of the aromatic compound degradation module encodes the operon benABCD for benzoate utilization, and another module, M00568, includes *cat* genes for catechol utilization.

### 3.6. Prediction of Virulence Factors

**Figure 6:**
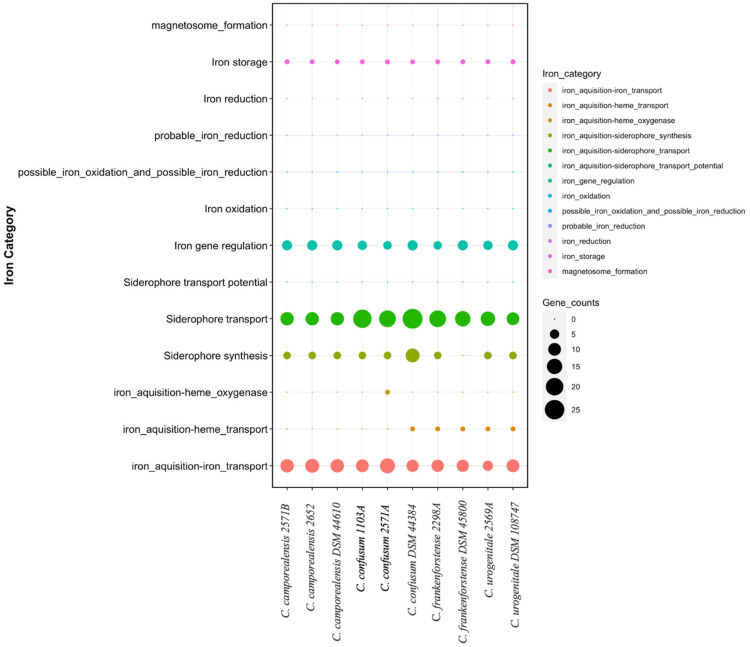
Dot-plot results of FeGenie software, which aids in determining iron-related pathways and genes in the genomes of the six *Corynebacterium* analyzed here. *Corynebacterium* species are mentioned on the x-axis, while iron-related gene groups are on the y-axis and shown by different colors.

A blast against VFDB revealed some *CdiLAM* genes involved in adherence in the genome of uterine *Corynebacterium* isolates, except in *Corynebacterium* sp. 335C (Supplementary Table S2). The exotoxin tox and pld genes were not found in all uterine *Corynebacterium* isolates, but the *dtxR* regulator gene was found in all species. The *fagABCD* genes involved in the ABC transporter of iron uptake were found in most species. In contrast, *hmuTUV* genes involved in heme transport were found in *Corynebacterium* sp 335C and *C. urogenitale* 2569A only (Supplementary Table S2). Deep analysis for iron-related genes was performed using the FeGenie tool, which revealed differences in iron uptake-related genes between species. The overall comparison is shown in Figure 6.

## 4. Discussion

Currently, the 95–96% ANI species boundary cutoff range is known as an equivalent to the 70% dDDH value. Nevertheless, during our routine identification of bacteria from a camel’s uterus with a history of conception failure, seven *Corynebacterium* strains were isolated. Of note, we found that strain 2569A, one of the seven *Corynebacterium* strains mentioned above, should be classified as a new *Corynebacterium* species, according to the 70% dDDH cutoff value, at the same time, strain 2569A should be classified as *C. urogenitale*, according to the current 95–96% OrthoANI cutoff value. Moreover, two species pairs of *Corynebacterium*-type strains showed conflicting classification results between their OrthoANI and dDDH values. Hence, what are the causes for this conflicting classification result? To clarify the above inconsistent classification results, the correlation between dDDH and OrthoANI from the 66 pairs of *Corynebacterium* species was assessed by a logarithmic regression model. We found that OrthoANI strongly correlates with dDDH values. However, a 70% dDDH value did not correspond to a 95–96% OrthoANI value but to an OrthoANI value of approximately 96.67%. Thus, the inconsistency above can be well clarified based on the above-mentioned correlation analysis. Therefore, we proposed that a 96.67% OrthoANI value could act as the cutoff for delineating *Corynebacterium* species.

For the past many years, the *16S rRNA* gene has emerged as a rapid reliable genetic approach for the identification of bacteria [43]. However, due to its limited resolution, this marker cannot reliably classify some bacteria to species level or provide robust phylogenetic relationships of certain species [44,45]. In this study, the limitation of *16S rRNA* gene analysis was also shown when we examined the pairwise similarity values of this gene for *Corynebacterium* validly published species. A pairwise sequence comparison revealed that 22 pairs of species exceeded the 98.7% cutoff values for species delineation. Thus, *16S rRNA* is not always reliable for *Corynebacterium* classification at the species level. Therefore, strains 2652, 2571B, 2569A, 1103A, 2571A, 2298A, and 335C were only assigned to the genus *Corynebacterium* based on the *16S rRNA* gene sequence similarity. Currently, taxonomists largely depend on phylogenetic tree inference to classify bacterial species based on the level of phylogenetic linkage [46]. There is proof that phylogenomic analysis outperforms phylogenetic analysis based on *16S rRNA* gene sequences in terms of resolution [47]. Consequently, we inferred the evolutionary position of uterine strains via a phylogenomic tree based on 244 single-copy core genes. Phylogenomic trees clearly clustered each uterine isolate to its closest type species, supported by high bootstrap values (100%).

According to the proposal by Chun et al. [23], any species having a ≥ 98.7% *16S rRNA* identity should be subjected to the ANI or dDDH approach. Consequently, the uterine strains 2652, 2571B, 2569A, 1103A, 2571A, and 2298A with 99% sequence similarity were classified using the ANI and dDDH approaches. The results revealed that strains 2652 and 2571B were classified as *C. camporealensis*. Strains 1103A, 2571A, 335C and strain 2298A, represent novel *Corynebacterium* species.

Among existing classification methods, gene content analysis is also at the heart of bacterial taxonomy. Here, we wanted to present the general genome feature and analyze gene content variation among uterine *Corynebacterium* strains. First, we assessed the level of dissimilarity in gene content between each strain of uterine *Corynebacterium* and its closely related type strain in order to determine if these differences can aid in species identification. This analysis was performed by counting the number of unshared genes between each uterine *Corynebacterium* strain and its closely related type strain. We found that the uterine *Corynebacterium* strain cannot be reliably classified into different species using gene content. This is due to the unexpected results, where a strain with 96% ANI showed a higher level of gene content diversity than a strain with 94% ANI. Our results are in agreement with a study where it was found that *Acinetobacter* cannot be reliably classified into different species using gene content [48]. The percentage of specific genes found in the study is similar to what was observed before in some *Corynebacterium* species [49]. Finally, a holistic comparison of the gene arrangement of uterine *Corynebacterium* strains with their closely related type strains unveiled large regions of gene synteny. Synteny breaks occurred in some regions by the strain-specific genes.

Secondly, to examine the dynamics of gene content differences in more detail, we investigated gene gain/loss (turnover) events for each uterine *Corynebacterium* strain through the phylogeny. We found a dynamic pattern of genome evolution marked by more gene gain than loss on the terminal branches. Our findings are similar in part to previous studies on the genus *Streptococcus*, whereas evolution in terminal branches was characterized by high gain [50]. Notably, the strain showing the greatest gene gain was strain 2571A (gain = 423, loss = 21), which may indicate that this species was changing or extending its niche. In contrast, the two strains of *C. urogenitale* from the uterus of cattle and camel showed similar turnover for the gene gain with a slight difference in the gene loss. Thus, gene turnover contributed to the genetic diversity of uterine species. Our findings are in agreement with a previous study [51]. The acquisition of foreign genes could convey new traits, which are necessary for virulence and adaptation into various niches and contribute to pathogen diversity [52,53]. We found a diverse pool of mobile genetic elements, including genomic islands (MGEs) and intact or remanent prophages, which indicates a high frequency of genetic transfer in uterine *Corynebacterium* strains. Therefore, the presence of MGEs facilitated the horizontal gene transfer and the high gene gain, which contributed to the observed variation in gene repertoire. The functional implications of these horizontally transferred elements resulted in the acquisition of different traits that resulted in enhancing the defense system by the acquisition of the CRISPR system, increasing virulence by increasing iron uptake and increasing nutrients inside the cell via transporters. Moreover, two potassium uptake systems were acquired, *KtrAB* and *KtrCD*, which are important for responses to osmotic stress [54]. Even though the specific traits required for uterine adaptation are not yet well defined, we believe that the aforementioned acquired traits might help uterine *Corynebacterium* adapt to the uterus.

Although uterine *Corynebacterium* strains share a similar ecology, they exhibit different functional profiles. This is probably due to the role of previous ecology and evolutionary processes, such as gene loss or gain, in shaping the functional profile. For instance, strains 2571A and 1103A seem to have a potential capacity to utilize benzoate as a carbon source and energy source. These findings are similar to what were observed before in the cow-associated *C. cystitidis* strain isolated from pyelonephritis cases [12]. The latter seems to have a potential capacity to utilize benzoate, which is present in cow urine, as a carbon source. This could reflect that the previous habitat of 2571A and 1103A was the urinary system. Furthermore, beta-glucosides, such as cellobiose, that result from the hydrolysis of plant cellulose are transported by the β-glucoside PTS transporter of strain 2571A; therefore, decaying vegetation can act as a source of carbon for soil bacteria. This suggests that strain 2571A has the potential to survive in soil via the utilization of decaying vegetation as a carbon source.

Finally, we wanted to determine the potential virulence capacity of uterine *Corynebacterium* isolates. Some of the potential virulence factors in VFDB were found in the genomes. The diphtheria toxin was not detected in the isolates, but its regulatory gene *dtxR* was found. Also, *C. pseudotuberculosis* pld toxin was not detected in the isolates. Iron acquisition is an important process for bacterial survival inside animal hosts because iron is hidden by the host. Thus, genes required for iron acquisition are called virulence factors. Among the virulence factors, *hmuVTU* genes involved in heme transport, *fagABCD* genes involved in iron uptake, and genes involved in adherence were found in some isolates. Thus, uterine *Corynebacterium* displayed different potentials for iron uptake and transport with strain 2298A, showing less capacity for iron uptake and transport. These findings suggest that the isolates of *Corynebacterium* from the uterus have varying potential pathogenicity capacities.

In conclusion, updating the ANI cutoff value to correlate with the dDDH cutoff value will enable using both ANI and dDDH approaches for the classification of *Corynebacterium* species. This gives more confidence in classification than using a single approach. This study demonstrated that the 96.67% OrthoANI value should be used in place of the generally accepted 95–96% ANI threshold for an accurate diagnosis of *Corynebacterium*, as the 95–96% ANI value does not always correlate with the 70% dDDH threshold. We isolated and classified for the first time from the uterus of camels *C. camporealensis*, *C. urogenitale*, and four novel *Corynebacterium* species, expanding the type of mammalian hosts from which these species have been isolated to date. Gene gain predominates as a source of variation in gene repertoire. Most such genes are gained by horizontal gene transfer driven by genomic islands and prophage. Finally, we provided the most updated phylogenomic tree to be used for inferring the evolutionary position of *Corynebacterium* species.

## Figures and Tables

**Table 1:** General genome features of uterine *Corynebacterium* strains and their related type strains.

Species	Isolate Source	GC [%]	Size (bp)	CDS	tRNA	Accession Number
*Corynebacterium* sp. 2298A	Camel uterus	71.2	2,569,049	2218	53	JASSPM000000000
*C. frankenforstense* DSM 45800	Cow milk	71.5	2,604,152	2163	52	NZ_CP009247.1
*C. urogenitale* 2569A	Camel uterus	60.1	2,353,837	2056	48	JASTZI000000000
*C. urogenitale* DSM 108747	Cow uterus	59.9	2,351,892	1994	48	NZ_CP045032
*C. camporealensis* DSM 44610	Sheep milk	59.4	2,451,810	2213	50	NZ_CP011311.1
*C. camporealensis* 2652	Camel uterus	59.4	2,396,631	2231	51	JASUCR000000000
*C. camporealensis* 2571B	Camel uterus	59.4	2,412,951	2277	50	JASUCQ000000000
*Corynebacterium* sp. 1103A	Camel uterus	65.3	2,429,847	2199	54	JASTTF000000000
*Corynebacterium* sp. 2571A	Camel uterus	65.6	2,648,610	2431	54	JASTTE000000000
*C. confusum* DSM 44384	Human plantar abscess		2.5 MB			NZ_CP047202.1
*Corynebacterium* sp. 335C	Camel uterus	73.7	2,733,887	2257	60	JASTTB000000000

**Table 2:** General features of the defense system, genomic island, and prophage.

Strain	CRISPR-Cas Subtype	RM Type	Prophage				Genomic Island		
			Intact	Incomplete	Size (Kb)	Total Genes	Number	Total Genes	Total Size (bp)
*Corynebacterium* sp. 2298A	-	-	-	3	24.9	26	14	108	131,205
*C. frankenforstense* DSM 45800	I-E	I	-	2	15.7	18	9	99	123,257
*C. urogenitale* 2569A	-	III	-	3	28.9	27	5	63	53,682
*C. urogenitale* DSM 108747	-	-	-	3	26.4	27	9	174	174,446
*Corynebacterium* sp. 1103A	-	III	-	1	13.9	19	14	129	136,169
*C. confusum* DSM44384	-	I and III	-	1	20.2	9	18	216	242,269
*Corynebacterium* sp. 2571A	I-E	I	1	4	116.2	123	20	326	261,524
*C. camporealensis* 2571B	I-E	I and III	-	1	4	7	11	147	133,482
*C. camporealensis* 2652	I-E	I	-	1	12.3	15	7	56	64,530
*C. camporealensis* DSM 44610	-	III	-	2	17	20	9	158	148,322

## Data Availability

Data are available on a publicly accessible GenBank website, and the accession number is mentioned in Table 1.

## Supplementary Materials

The following supporting information can be downloaded at https://www.mdpi.com/article/10.3390/vetsci11120661/s1, Table S1: values for OrthoANI, dDDH, and 16S rRNA from a pairwise comparison of *Corynebacterium* species; Table S2: virulence factors predicted in *Corynebacterium* species from camel uteri.
