# Supplementary material for: Updating the Relationship Between the Threshold Value of Average Nucleotide Identity and Digital DNA–DNA Hybridization for Reliable Taxonomy of Corynebacterium
Source: Vet Sci. 2024 Dec 17;11(12):661. doi: 10.3390/vetsci11120661 (PMC11680202; doi:10.3390/vetsci11120661)
Supplement: Supplementary file 1 [file vetsci-11-00661-s001.zip › vetsci-3309608-supplementary.pdf]

Supplementary Table S1. values for OrthoANI, dDDH and *16S rRNA* form pairwise comparison of *Corynebacterium* species. Dash (-) indicate short *16S rRNA* sequence. Therefore, not included in the analysis

**Species**	**Species**	**dDDH %**	**OrthoANI %**	***16S rRNA* %**
C. belfantii FRC0043	*C. belfantii* 07 1	97	99.67	100
C. belfantii FRC0043	*C. belfantii* 01 16	97.5	99.67	100
C. amycolatum FDAARGOS 1108	*C. amycolatum* FDAARGOS 1189	94.4	99.25	100
C. camporealensis 2571B	*C. camporealensis* DSM 44610	88.5	98.72	99.67
C. camporealensis 2652	*C. camporealensis* DSM 44610	85.8	98.44	99.15
C. diphtheriae bv mitis str NCTC3529	*C. diphtheriae* bv mitis str ISS 3319	83.9	98.25	99.87
C. afermentans subsp afermentans DSM 44280	*C. afermentans* ACRQQ	79	97.71	99.87
C. afermentans subsp afermentans DSM 44280	*C. afermentans* SCPM O B 9437	79.5	97.7	99.87
C. afermentans subsp afermentans DSM 44280	*C. afermentans* MGYG HGUT 01701	77.8	97.57	99.74
C. afermentans subsp afermentans DSM 44280	*C. afermentans* ACRPV	77.5	97.52	99.67
C. accolens DSM 44278	*C. segmentosum* NCTC934	74.1	97.05	99.41
C. urogenitale 2569A	*C. urogenitale* DSM 108747	69.4	96.58	99.54
C. ureicelerivorans IMMIB RIV-2301	*C. mucifaciens* ATCC 700355	65.7	95.9	99.01
C. belfantii FRC0043	*C. diphtheriae* bv mitis str ISS 3319	61.7	95.26	99.47
C. pilbarense CCUG 57942	*C. afermentans* subsp *lipophilum* CCUG 32105	57.8	94.61	98.99
C. afermentans subsp *afermentans* DSM 44280	*C. afermentans* subsp *lipophilum* DSM 44282	57.1	94.59	99.08
Corynebacterium sp 2571A	*C. confusum* DSM 44384	54.9	94.26	99.74
C. tuberculostearicum FDAARGOS 1117	*C. marquesiae* c19Ua 121	55.6	94.15	99.54
Corynebacterium sp 2298A	*C. frankenforstense* DSM 45800	51.3	94.03	99.28
Corynebacterium sp 1103A	*C. confusum* DSM 44384	54.1	94.01	99.74
C. kroppenstedtii DSM 44385	*C. pseudokroppenstedtii* MC 17X	49.9	93.18	99.8
C. rouxii FRC0190	*C. diphtheriae* bv mitis str ISS 3319	49.1	92.85	99.15
C. kroppenstedtii DSM 44385	*C. parakroppenstedtii* MC 15	47.6	92.61	99.56
C. evansiae c8Ua 174	*C. jeikeium* K411	44.5	91.84	-
C. sanguinis CCUG 58655	*C. lipophiloflavum* DSM 44291	45.1	91.74	98.69
C. lehmanniae c8Ua 144	*C. afermentans* subsp *lipophilum* CCUG 32105	44.3	91.67	98.18
C. silvaticum PO100 5	*C. ulcerans* 809	41.2	90.52	99.67
C. meridianum CCM 9186	*C. antarcticum* P6129	38.8	89.8	99.47
C. tuberculostearicum FDAARGOS 1117	*C. yonathiae* c21Ua 68	36.4	88.9	99.54
C. hesseae c19Ua 109	*C. aurimucosum* ATCC 700975	36	88.9	99.79

C. pelargi 136 3	*C. pseudopelargi* 812CH	35.3	88.69	99.8
C. macginleyi 160811	*C. accolens* DSM 44278	35.8	88.5	98.88
C. curieae c8Ua 181	*C. marquesiae* c19Ua 121	34.6	88.3	99.87
C. atrinae JCM 19266	*C. testudinoris* DSM 44614	34	87.99	98.7
C. auris DSM 44122	*C. timonense* 5401744	32.6	87.3	97.75
C. comes 2019	*C. marinum* DSM 44953	32.5	87.2	98.42
C. macclintockiae c9Ua 112	*C. evansiae* c8Ua 174	30.3	86.95	-
C. fournieri Marseille-P2948	*C. lehmanniae* c8Ua 144	31.2	86.69	98.25
C. freneyi FDAARGOS 1426	*C. hansenii* DSM 45109	30.2	86.39	98.88
C. marambiense P5848 1	*C. antarcticum* P6129	30.7	86.33	99.67
C. xerosis GS 1	*C. hansenii* DSM 45109	29.5	86.13	99.01
C. humireducens NBRC 106098	*C. pollutisoli* VDS11	29.3	85.94	98.62
C. tuscaniense DNF00037	*C. meitnerae* c8Ua 172	28.8	85.38	99.37
C. pygosceleis P7374 1	*C. meridianum* CCM 9186	28.8	85.38	99.28
C. silvaticum PO100 5	*C. pseudotuberculosis* MEX29	28.7	85.13	99.47
C. wankanglinii zg-915	*C. ureicelerivorans* IMMIB RIV 2301	27.7	84.49	97.48
C. glutamicum SCgG2	*C. suranareeae* N24	27.4	84.37	98.56
C. minutissimum NCTC10288	*C. singulare* IBS B52218	27.4	84.19	99.27
C. striatum FDAARGOS 1115	*C. simulans* Wattiau	28.1	83.8	98.42
C. urinipleomorphum Marseille-P2799T	*C. appendicis* DSM 44531	26.2	83.81	98.25
C. propinquum FDAARGOS 1112	*C. pseudodiphtheriticum* DSM 44287	28.8	83.8	99.41
C. kalidii LD5P10	*C. glyciniphilum* AJ 3170	25.8	83.35	98.3
C. megadyptis subsp *dunedinense*7B	*C. ciconiae* DSM 44920	25.4	82.99	98.93
C. jeddahense DSM 45997	*C. lehmanniae* c8Ua 144	25.6	82.86	97.62
C. mycetoides DSM 20632	*C. lipophiloflavum* DSM 44291	25.1	82.15	97.5
C. gottingense DSM 103494	*C. imitans* NCTC13015	24.4	82.08	98.22
C. coyleae DSM 44184	*C. lujinxingii* zg 917	24.4	82.08	98.81
C. oculi NML 130210	*C. mastitidis* DSM 44356	24.2	82.06	98.21
C. glutamicum SCgG2	*C. crudilactis* JZ16	24.7	82	98.03
C. terpenotabidum Y-11	*C. variabile* NBRC 15286	23.9	81.67	98.62
C. faecale DSM 45971	*C. efficiens* YS 314	24.2	81.44	98.1
C. sanguinis CCUG 58655	*C. qintianiae* MC1420	23.7	81.19	98.68
C. stationis 622DSM 20302	*C. ammoniagenes* MGYG HGUT 01533	23	80.6	98.16
C. stationis 622DSM 20302	*C. casei* LMG S 19264	23.3	80.6	98.75
C. oculi NML 130210	*C. lowii* NML 130206T	22.7	80.57	97.75
C. glucuronolyticum FDAARGOS 1111	*C. pyruviciproducens* ATCC BAA 1742	26	80.22	96.86
C. nuruki S6-4	*C. variabile* NBRC 15286	22.7	79.99	97.96
C. halotolerans DSM 44683	*C. pollutisoli* VDS11	22.4	79.36	96.06
C. cystitidis NCTC11863	*C. pilosum* NCTC11862	21.2	78.88	95.07

C. mycetoides DSM 20632	*C. liangguodongii* 2184	21.2	78.58	97.76
C. resistens DSM 45100	*C. auriscanis* DSM 44609	21.9	78.29	99.39
C. gerontici W8	*C. pseudopelargi* 812CH	22.1	77.7	98.48
C. frankenforstense DSM 45800	*C. atypicum* R2070	22.1	77.37	94.59
Corynebacterium sp 335C	*C. sphenisci* DSM 44792	21.3	77.12	97.51
C. amycolatum FDAARGOS 1108	*C. lactis* RW2 5	22.7	76.96	98.29
C. maris DSM 45190	*C. guangdongense* DSM 107476	21.1	76.81	94.62
C. genitalium ATCC 33030	*C. meitnerae* c8Ua 172	20.9	76.76	98.39
C. falsenii FDAARGOS 1493	*C. evansiae* c8Ua 174	22.8	76.57	
C. kalinowskii 1959	*C. hindlerae* NML 93 0612	19.8	76.44	96.71
C. callunae DSM 20147	*C. deserti* GIMN1.010	21.3	76.27	97.18
C. riegelii PUDD 83A45	*C. glaucum* DSM 30827	20.6	76	97.02
C. capitovis DSM 44611	*C. timonense* 5401744	20	75.81	96.34
C. bovis 4826	*C. kalidii* LD5P10	21.4	75.8	96.84
C. massiliense DSM 45435	*C. confusum* DSM 44384	20.8	75.7	96.18
C. yudongzhengii 2183	*C. doosanense* DSM 45436	19.8	75.39	94.32
C. uberis 18M0132	*C. mastitidis* DSM 44356	21.3	74.91	95.96
C. camporealensis DSM 44610	*C. tuberculostearicum* FDAARGOS 1117	21.2	74.9	95.93
C. aquatimens DSM 45632	*C. appendicis* DSM 44531	23.5	74.86	97.24
C. frankenforstense DSM 45800	*C. otitidis* ATCC 51513	21.1	74.84	91.41
C. pseudogenitalium CCUG 27540	*C. imitans* NCTC13015	21	74.69	95.2
C. anserum 23H37-10	*C. urogenitale* LMM 1652	22.6	74.63	98.69
C. flavescens OJ8	*C. simulans* Wattiau	21.3	74.5	96.21
C. suicordis DSM 45110	*C. urogenitale* LMM 1652	21.9	74.34	96.35
C. occultum 2039	*C. efficiens* YS 314	21.4	74.26	95.21
C. endometrii LMM-1653	*C. casei* LMG S 19264	22.2	74.3	95.93
C. lubricantis DSM 45231	*C. pilosum* NCTC11862	19.1	74.19	95.83
C. alimapuense VA37-3	*C. testudinoris* DSM 44614	19.3	73.59	96.02
C. epidermidicanis DSM 45586	*C. hindlerae* NML 93 0612	21.9	73.22	96.65
C. urealyticum NCTC12011	*C. heidelbergense* DSM 104638	22.1	73.14	94.97
C. lizhenjunii ZJ-599	*C. phocae* M408 89	22.4	73.1	96.57
C. aquilae dsm 44791	*C. argentoratense* DSM 44202	25.5	72.88	96.92
C. sphenisci DSM 44792	*C. sputi* DSM 45148	20.2	72.8	95.4
C. zhongnanshanii zg-320	*C. auriscanis* DSM 44609	23.9	72.55	96.78
C. freiburgense DSM 45254	*C. canis* CCUG 58627	17.8	72.43	97.39
C. vitaeruminis DSM 20294	*C. gerontici* W8	22.4	72.41	96.58
C. tapiri LMG 28165	*C. uterequi* DSM 45634	20.7	72.37	95.36
C. durum F0235	*C. canis* CCUG 58627	21.2	71.3	92.33
C. felinum CCUG 39943	*C. mustelae* DSM 45274	23.5	71.13	96.56

C. spheniscorum J11	*C. uropygiale* JCM 32435	21.8	70.7	95.64
C. poyangense 4H37-19	*C. uropygiale* JCM 32435	18.5	70.5	96.93
C. matruchotii ATCC 14266	*C. mustelae* DSM 45274	21.7	70.13	94.15
C. ulceribovis DSM 45146	*C. lactis* RW2 5	21	69.91	96.3
C. choanae 200CH	*C. aquilae* dsm 44791	25.9	69.46	95.75
C. kutscheri DSM 20755	*C. mustelae* DSM 45274	26.7	69.15	95.44
C. renale NCTC7448	*C. caspium* DSM 44850	22.4	68.7	95.04

Supplementary Table S2. Virulence factors predicted in *Corynebacterium* species from camel uterus. All virulence factors of *Corynebacterium* predicted in VFDB were listed in the table. Dash (–) indicate absence of the virulence factors.

**Virulence factors**	**Related genes**	*Corynebacterium sp* 1103A	*Corynebacterium* sp 2571A	*Corynebacterium* sp 335C	*C. camporealensis* 2571B	*C. camporealensis* 2652	*Corynebacterium* sp 2298A	*C. urogenitale* 2569A
**Adherence**								
CdiLAM	*emb*	*emb*	*emb*	-	*emb*	*emb*	*emb*	*emb*
	aftB	*aftB*	*aftB*	-	*aftB*	*aftB*	*aftB*	-
	mptC	-	-	-	-	-	-	-
	mptD	-	-	-	-	-	-	-
Collagen-binding protein	*cbpA*	-	-	-	-	-	-	-
SpaA-type pili	*spaA*	*srtA*	*srtA*	-	*srtA*	*srtA*	*srtA*	*srtA*
	srtA	-	-	-	-	-	-	-
	spaB	-	-	-	-	-	-	-
	spaC	-	-	-	-	-	-	-
SpaD-type pili	*srtB*	-	-	-	-	-	-	-
	spaD	-	-	-	-	-	-	-
	srtC	-	-	-	-	-	-	-
	spaE	-	-	-	-	-	-	-
	spaF	-	-	-	-	-	-	-
SpaH-type pili	*spaI*	-	-	-	-	-	-	-
	srtE	-	-	-	-	-	-	-
	srtD	-	-	-	-	-	-	-
	spaH	-	-	-	-	-	-	-
	spaG	-	-	-	-	-	-	-
Surface-anchored pilus proteins	*sapA*	-	-	-	-	-	-	-
	sapD	-	-	-	-	-	-	-

	sapE	-	-	-	-	-	-	-
**Exotoxin**								
DT	tox	-	-	-	-	-	-	-
Phospholipase D	pld	-	-	-	-	-	-	-
**Nutritional/Metabolic factor**								
ABC transporter	*fagC*	*fagC*	*fagC*	*fagC*	-	*fagC*	*fagC*	-
	fagB	*fagB*	*fagB*	*fagB*	-	*fagB*	*fagB*	*fagB*
	fagA	*fagA*	*fagA*	*fagA*	-	*fagA*	*fagA*	*fagA*
	fagD	*fagD*	-	*fagD*	*fagD*	*fagD*	*fagD*	*fagD*
ABC-type heme transporter	*hmuT*	-	-	*hmuT*	-	-	-	*hmuT*
	hmuU	-	-	*hmuU*	-	-	-	*hmuU*
	hmuV	-	-	*hmuV*	-	-	-	*hmuV*
Ciu iron uptake and siderophore biosynthesis system	*ciuA*	-	-	-	-	-	-	-
	ciuB	-	-	-	-	-	-	-
	ciuC	-	-	-	-	-	-	-
	ciuD	-	-	-	-	-	-	-
	ciuE	-	-	-	-	-	-	-
Siderophore-dependent iron uptake system	*irp6A*	-	-	-	-	-	-	-
	irp6B	-	-	-	-	-	-	-
	irp6C	-	-	-	-	-	-	-
**Regulation**								
Diphtheria toxin repressor *DtxR*	*dtxR*	*dtxR*	*dtxR*	*dtxR*	*dtxR*	*dtxR*	*dtxR*	*dtxR*
